# Photobiomodulation associated with sclerotherapy for treatment of hemangioma in the hard palate

**DOI:** 10.1590/1677-5449.200189

**Published:** 2021-07-09

**Authors:** Matheus Sampaio de Oliveira, Maurilio Araujo Pegas, Larissa Pavan de Deus, Paula Carolina de Souza Chandretti, Eduardo Machado Vilela

**Affiliations:** 1 Universidade Federal de Juiz de Fora – UFJF, Juiz de Fora, MG, Brasil.; 2 Associação Brasileira de Odontologia – Regional Juiz de Fora – ABO/JF, Juiz de Fora, MG, Brasil.

**Keywords:** hemangioma, sclerotherapy, low-level light therapy, oral medicine

## Abstract

Vascular changes frequently involve the head and neck region and hemagioma is the most common. A 61-year-old female patient complained of severe pain in the hard palate. A purple lesion was found, measuring 1.5 cm, sensitive to palpation, and with a history of hemorrhage. The patient was fully edentulous and her upper denture compressed the lesion site. Diascopy confirmed the lesion’s vascular origin. A diagnostic hypothesis of hemangioma was raised. In the first session, red laser light (660nm) was applied at 4 points around the lesion, with 0.5 J at each point, in order to obtain analgesia and trigger the repair process. The upper denture was also relined. In the second session, 2 mL of 5% monoethanolamine oleate was applied. After 14 days, total regression of the lesion was observed. Dental surgeons must be able to recognize, diagnose and treat vascular lesions in the oral cavity.

## INTRODUCTION

Vascular anomalies have high incidence in the head and neck region. Around 60% of hemangiomas and other vascular lesions occur in these anatomic sites. The sites most affected in the oral cavity are the lips, tongue, buccal mucosa, and palate.^1^ Classification of these pathologies is controversial, because there is a long list of variables that must be considered at the point of diagnosis, such as the clinical, histopathological, and biological features of the lesion. Currently, these anomalies are classified as tumors or vascular malformations.^2^ Hemangiomas may be considered benign vascular tumors that develop during childhood, but which rarely affect the adults and the elderly. In contrast, some authors consider hemangioma to be a widely-applicable clinical term to refer to endothelial malformations. Considering clinical features, they are generally asymptomatic, size can range from a few millimeters to several centimeters, they may be flat or raised, sessile or pedunculated, and are soft on palpation. Color is related to the site of the lesion, and to tissue depth, and can vary from red to purple.^3^

Diagnosis of oral vascular lesions is founded on a combination of their clinical characteristics and the history provided by the patient. In some cases, certain supplementary tests can guide diagnosis and treatment planning. These include diascopy, needle aspiration, and imaging exams. After confirmation of vascular origin, the hemodynamic characteristics of the lesion are also needed for treatment planning.^3^

Esthetic issues tend to be the main complaint among people suffering from vascular conditions. There may also be pain, ulceration, problems with masticatory function, airway obstruction, bleeding, tissue deformation, and interference with occlusion.^2^^,^^4^^,^^5^ Treatment options include surgical removal, systemic corticosteroids, cryotherapy, embolization, radiotherapy, high power laser, and sclerotherapy (SCT).^6^^,^^7^ It should be pointed out that the choice of treatment depends on a set of factors such as size, location, and hemodynamic characteristics.^3^

Since SCT is a noninvasive technique with high curative efficacy (70-100%), it can be considered a good technique for vascular anomaly cases. The mechanism of action of SCT is based on substitution of the vascular component by a membrane of fibrous tissue in response to an inflammatory process. Considering its action via inflammatory mechanisms, photobiomodulation (PTBM) can play a supportive therapeutic role because of its direct action on cellular energy metabolism, changing inhibition/release of cytoplasmic mediators. It is notable that, of the sclerosing agents available, ethanolamine oleate stands out for its low toxicity and excellent effectiveness under different concentrations. However, there is no standard protocol established for its administration and it should be handled with great care.^2^^,^^3^^,^^8^^-^^10^

The objective of this study is to report on a case in which a vascular anomaly in the palate associated with ill-fitting full dentures was treated using PTBM and SCT.

The Research Ethics Committee approved this study (decision number 4.810.099).

## PART I - CLINICAL SITUATION

**Figure 1:**
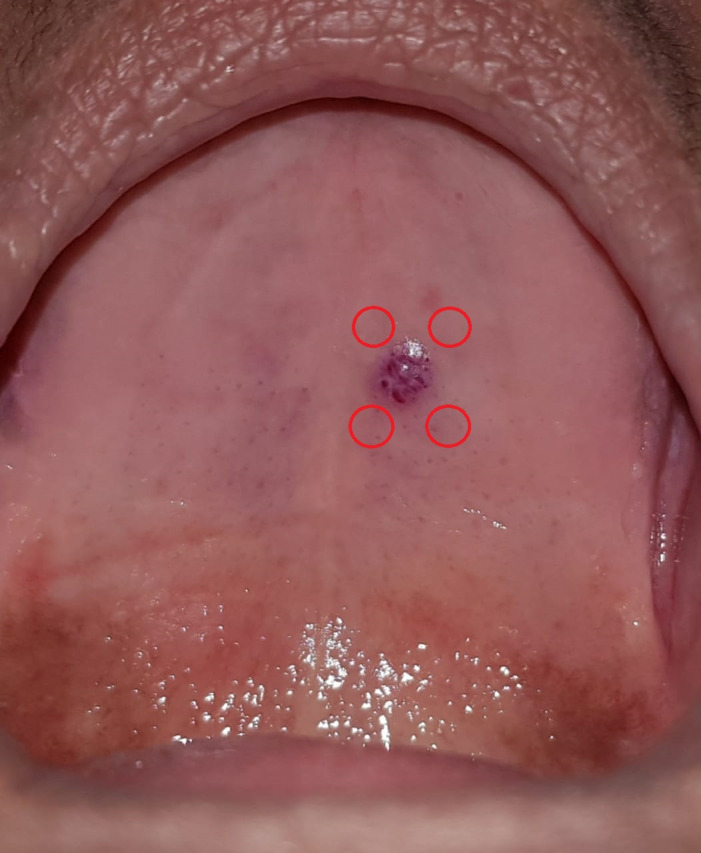
Initial intraoral view, showing the purple lesion of the hard palate and the points where Photobiomodulation (PTBM) was administered.

The patient was a 61-year-old female, with no history of systemic conditions, who presented at the Stomatology Department of the Faculty of Dentistry, Universidade Federal de Juiz de Fora (UFJF), MG, Brazil, complaining of moderate to intense pain in the hard palate. An extraoral examination detected nothing of note. The intraoral examination revealed a purple lesion of the hard palate with blood accumulated in its interior, approximately 1.5 cm in size, sensitive to palpation, with a history of bleeding, and onset 3 months previously (Figure 1). The patient was fully edentulous and wore both upper and lower dentures. The upper prosthesis had a suction chamber that compressed the lesion site.

## PART II - WHAT WAS DONE

A diascopy maneuver confirmed the lesion’s vascular origin, after local ischemia. A clinical diagnostic hypothesis of hemangioma was raised. Since the lesion was painful and had been bleeding, red laser light with a wavelength of 660 nm was administered at the first session, with power set at 100 mW and a spot area of 3 mm^2^ (Laser Duo Portátil^®^, MMO Ltda., São Carlos, Brazil), irradiating four points around the lesion (see Figure 1), administering 0.5 J at each point in order to achieve analgesia and trigger the healing process. The upper denture was also relined using soft resin in place of the suction chamber and the sclerotherapy procedure was scheduled.

**Figure 2:**
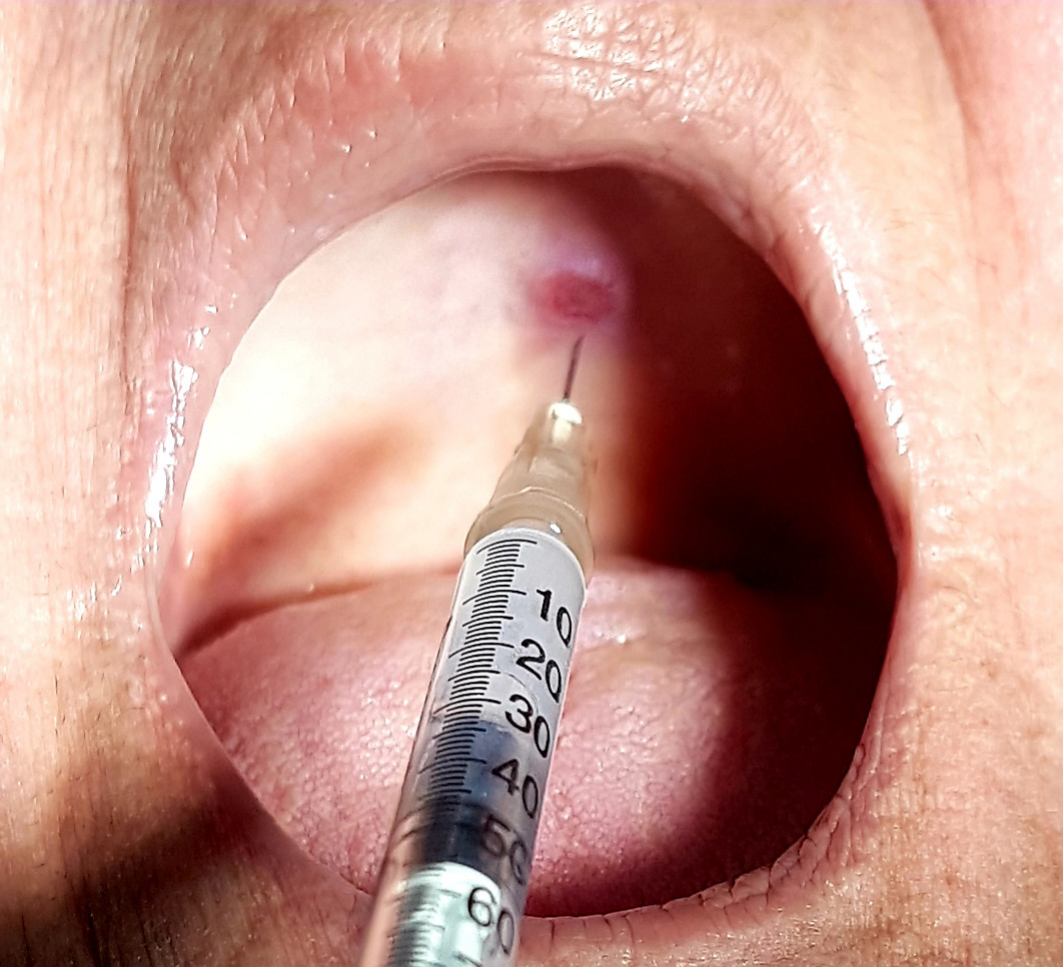
Application of ethanolamine oleate 5% around the lesion.

At the second session, the patient reported significant improvement in pain. Her blood pressure was measured at 120x70 mmHg. She was then given anesthesia with lidocaine 2% and epinephrine 1:100,000 around the lesion and 2 mL of monoethanolamine oleate 5% was administered at the base (Ethamolin^®^, Zest Pharmaceutical Ltda., Rio de Janeiro, Brazil) as shown in Figure 2.

**Figure 3:**
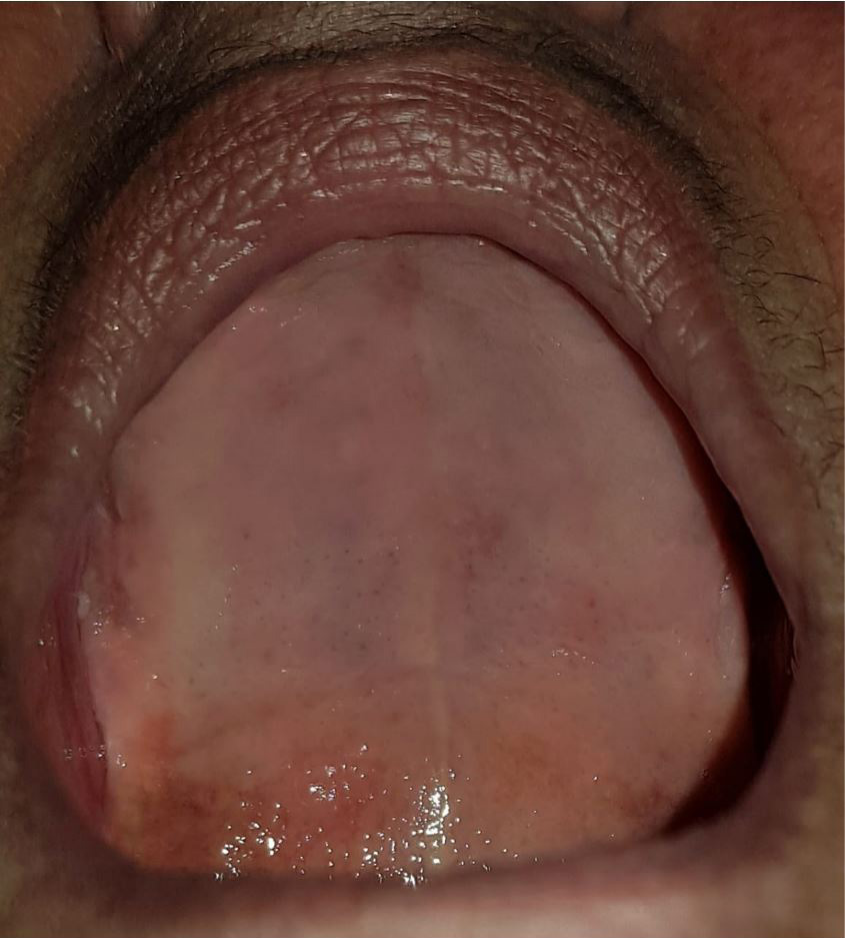
Intraoral view 14 days after administration, showing total regression of the lesion.

After 14 days, total regression of the lesion was observed (Figure 3). The patient was referred to be fitted for new dentures and is in outpatients follow-up.

## DISCUSSION

Hemangiomas are benign vascular lesions. Incidence is highest soon after birth or in early childhood. However, some cases develop in adulthood, with greater frequency among females.^11^ These lesions can affect the whole of the body, but when the oral cavity is involved, the sites most affected are the lips, tongue, buccal mucosa, and palate.^5^^,^^11^^-^^13^ The clinical case reported here shares these characteristics, since the patient was an adult female with a lesion involving the palate.

With regard to the origin of these lesions, they can be associated with congenital or traumatic factors,^11^ as observed in the present case, in which the patient’s upper denture was compressing the lesion site. Diagnosis can be made simply and safely by combining the findings of patient history, physical examination, and semiotic maneuvers such as diascopy, which helps to establish differential diagnosis.^3^ The clinical features of these lesions manifest depending on their depth and location. Color can vary from red to purple, margins are well-defined, consistency may be elastic or fibrous, and the surface may be smooth or irregular.^3^^,^^12^^,^^14^ Size varies from millimeters to centimeters.^3^ They exhibit progressive growth and are prone to unexpected and difficult to control bleeding, in response to traumatism of the lesion site.^11^ Additionally, pain, ulceration, problems with masticatory function, airway obstruction, and tissue deformation may also be present.^2^

When choosing the type of treatment for hemangiomas, certain attributes should be taken into consideration, such as size, location, and duration of the lesion, age of the patient, hemodynamics (by observation of blood flow), and technical feasibility.^3^^,^^15^ Treatment options include surgical removal, administration of systemic corticosteroids, cryotherapy, embolization, radiotherapy, laser therapy, and SCT.^7^ In the case described here, treatment was based on a combination of low intensity laser therapy and SCT.

Low intensity laser therapy, also known as PTBM, uses low light levels in the red or infrared wavelengths. This acts on damaged tissues, provoking cure, remodeling, and/or reduction of inflammation, which induces analgesia.^16^ Advantages of laser therapy described in the literature include an absence of side effects, safe treatment of patients with systemic compromise, minimally invasive approach, painlessness, and short duration of sessions.^8^^,^^10^^,^^17^ The analgesic effect of laser therapy was observed in this case, in which the patient who had been suffering acute pain, reported significant improvement in this symptom after the procedure.

In turn, SCT is an effective technique for treatment of hemangiomas,^3^^,^^15^ but there are no specific protocols.^13^ The sclerosing agent can be an oleic acid derivative, with hemostatic properties. Its action is the result of an inflammatory response, involving substitution of the vascular component with fibrous tissue.^3^^,^^15^ Monoethanolamine oleate 5% produced satisfactory results, with good regression of the lesion and minimal morbidity and adverse effects.

It is necessary to elucidate the origins of vascular lesions of the oral cavity, such as hemangiomas. Dental surgeons must be able to recognize, diagnose and treat them, or make the appropriate referrals, when necessary. Treatment with SCT proved highly effective, minimally invasive, and safe and provoked a rapid response. The combination with PTBM can be employed with the aim of provoking analgesia and initiating the healing process.
