# Supplementary material for: Photobiomodulation associated with sclerotherapy for treatment of hemangioma in the hard palate
Source: J Vasc Bras. 2021 Jul 9;20:e20200189. doi: 10.1590/1677-5449.200189 (PMC8276641; doi:10.1590/1677-5449.200189)
Supplement: Translation

# Translation: Fotobiomodulação associada à escleroterapia no tratamento de hemangioma em palato duro

**Authors:** Matheus Sampaio de Oliveira, Maurilio Araujo Pegas, Larissa Pavan de Deus, Paula Carolina de Souza Chandretti, Eduardo Machado Vilela

**Affiliations:** 1 Universidade Federal de Juiz de Fora – UFJF, Juiz de Fora, MG, Brasil.; 2 Associação Brasileira de Odontologia – Regional Juiz de Fora – ABO/JF, Juiz de Fora, MG, Brasil.

**Keywords:** hemangioma, escleroterapia, *laser* de baixa intensidade, medicina bucal

## Abstract

As alterações vasculares ocorrem frequentemente em região de cabeça e pescoço, sendo o hemangioma a mais comum. Paciente do sexo feminino, 61 anos, queixou-se de dor intensa em palato duro. Notou-se lesão arroxeada, de 1,5 cm, sensível à palpação e com histórico de hemorragia. A paciente era edêntula total, e a prótese total superior comprimia o local da lesão. Foi realizada a vitropressão, confirmando a origem vascular. A hipótese diagnóstica foi de hemangioma. Na primeira sessão, aplicou-se o laser vermelho (660 nm) em quatro pontos ao redor da lesão, sendo 0,5 J em cada ponto afim de se obter analgesia e iniciar o processo de reparo, além do reembasamento da prótese total superior. Na segunda sessão, foi feita aplicação de 2 mL de oleato de monoetanolamina 5%. Após 14 dias, observou-se regressão total da lesão. Os cirurgiões-dentistas devem estar aptos a reconhecer, diagnosticar e tratar as lesões vasculares em cavidade oral.

## INTRODUÇÃO

As anomalias vasculares possuem alto índice de ocorrência nas regiões de cabeça e pescoço. Cerca de 60% dos hemangiomas e outras lesões vasculares ocorrem nesses sítios anatômicos. Em relação à cavidade oral, os locais mais afetados são os lábios, a língua, a mucosa jugal e o palato^1^. Essas patologias possuem uma classificação controversa, pois há uma grande gama de informações que devem ser consideradas no momento do diagnóstico, tais como os aspectos clínicos, histopatológicos e biológicos da lesão. Atualmente, essas anomalias são classificadas em tumores ou malformação vascular^2^. Os hemangiomas podem ser considerados tumores vasculares benignos que possuem seu desenvolvimento na infância, mas que, raramente, podem afetar idosos e adultos. Por outro lado, alguns autores consideram hemangioma um termo clínico de amplo uso, referente a malformações endoteliais. Considerando os aspectos clínicos, geralmente são assintomáticos, o tamanho pode variar de alguns milímetros a vários centímetros, podem ser planos ou elevados, sésseis ou pedunculados e de consistência mole à palpação. A coloração está relacionada ao local da lesão, bem como à profundidade tissular, podendo variar entre o vermelho e o roxo^3^.

O diagnóstico de lesões vasculares orais é construído através da união das características clínicas com o histórico da lesão fornecido pelo paciente. Em determinados casos, alguns exames complementares podem orientar no diagnóstico e no plano de tratamento. Entre eles, destacam-se a manobra de vitropressão, a punção aspirativa e os exames de imagem. Após a confirmação da origem vascular, outro ponto necessário no momento da elaboração do plano de tratamento são as características hemodinâmicas da lesão^3^.

Os indivíduos afetados por distúrbios vasculares costumam apresentar como queixa principal questões estéticas. Além disso, dor, ulceração, problemas na função mastigatória, obstrução das vias aéreas, sangramento, deformação tecidual e interferências oclusais também podem estar presentes^2^^,^^4^^,^^5^. As opções de tratamento incluem remoção cirúrgica, corticosteroides sistêmicos, crioterapia, embolização, radioterapia, *laser* de alta potência e escleroterapia (ESCT)^6^^,^^7^. Vale salientar que a escolha do tratamento depende de um conjunto de fatores como tamanho, localização e características hemodinâmicas^3^.

Por ser uma técnica não invasiva e de alta efetividade curativa (70-100%), a ESCT pode ser considerada uma boa abordagem em casos de anomalias vasculares. O mecanismo de ação utilizado pela ESCT baseia-se na substituição do componente vascular por uma membrana de tecido fibroso em resposta a um processo inflamatório. Considerando a ação por meio de mecanismos inflamatórios, a fotobiomodulação (FTBM) pode desempenhar papel de suporte terapêutico em razão da sua ação direta sobre o metabolismo energético celular, alterando a inibição/liberação de mediadores citoplasmáticos. Vale salientar que, entre os agentes esclerosantes, o oleato de etanolamina destaca-se por sua baixa toxicidade e excelente efetividade sob diferentes concentrações. Entretanto, não há um protocolo padrão estabelecido acerca de sua aplicação, e seu manejo deve ser realizado de maneira bastante criteriosa^2^^,^^3^^,^^8^^-^^10^.

O objetivo deste estudo é apresentar um relato de caso em que foi realizado o tratamento de uma alteração vascular na região palatal associado ao uso de prótese total mal-adaptada através da terapia da FTBM com ESCT.

## PARTE I – SITUAÇÃO CLÍNICA

Paciente do sexo feminino, 61 anos, sem histórico de alterações sistêmicas, compareceu ao Serviço de Estomatologia da Faculdade de Odontologia da Universidade Federal de Juiz de Fora (UFJF) queixando-se de dor moderada à intensa em palato duro. Ao exame extraoral, não foram constadas alterações. Ao exame intraoral, notou-se lesão arroxeada em palato duro com coleção sanguínea em seu interior, de aproximadamente 1,5 cm, sensível à palpação, com histórico de hemorragia e tempo de evolução de 3 meses (Figura 1). A paciente era edêntula total e utilizava prótese total superior e inferior, sendo que a superior possuía câmara de sucção que comprimia o local da lesão.

**Figura 1:** Vista intraoral inicial evidenciando lesão arroxeada em palato duro e os pontos de aplicação da fotobiomodulação (FTBM).

## PARTE II – O QUE FOI FEITO

Foi realizada a manobra de vitropressão, confirmando a origem vascular após a ocorrência de isquemia na região. A hipótese diagnóstica clínica foi de hemangioma. Devido ao quadro de dor e de hemorragia, na primeira sessão aplicou-se o laser vermelho de comprimento de onda de 660 nm, potência de 100 mW e área do spot de 3 mm^2^ (Laser Duo Portátil^®^, MMO Ltda., São Carlos, Brasil) em quatro pontos ao redor da lesão (conforme Figura 1), sendo 0,5 J em cada ponto afim de se obter analgesia e o início do processo de reparo. Foi feito também o reembasamento da prótese total superior com resina *soft* no local da câmara de sucção e agendamento para realização do procedimento de escleroterapia.

Na segunda sessão, foi relatada pela paciente uma melhora significativa da dor. A pressão arterial foi aferida em 120x70 mmHg. Foi executada, então, anestesia com lidocaína 2% com epinefrina 1:100.000 ao redor da lesão e foram aplicados em sua base 2 mL de oleato de monoetanolamina 5% (Ethamolin^®^, Zest Farmacêutica Ltda., Rio de Janeiro, Brasil) conforme Figura 2.

**Figura 2:** Aplicação de oleato de etanolamina 5% na periferia da lesão.

Após 14 dias, observou-se regressão total da lesão (Figura 3). A paciente foi encaminhada para a confecção de novas próteses e permanece em acompanhamento ambulatorial.

**Figura 3:** Vista intraoral após 14 dias da aplicação evidenciando total regressão da lesão.

## DISCUSSÃO

Os hemangiomas são lesões vasculares benignas. A maior incidência ocorre após o nascimento ou na primeira infância. Porém, alguns casos desenvolvem-se na fase adulta, acometendo, com maior frequência, o sexo feminino^11^. A lesão pode se estender por toda a extremidade corpórea, mas, quando afeta a cavidade bucal, os locais mais envolvidos são lábios, língua, mucosa jugal e palato^5^^,^^11^^-^^13^. O caso clínico relatado compartilha dessas características, uma vez que se refere à paciente adulta, do sexo feminino, com lesão envolvendo a região do palato.

Quanto à origem das lesões, pode estar associada a fatores congênitos ou traumáticos^11^, conforme observado no presente caso, em que a prótese total superior da paciente comprimia o local da lesão. O diagnóstico pode ser estabelecido de forma simples e segura pela associação entre anamnese, exame físico e manobras semiotécnicas como a vitropressão, a qual auxilia no estabelecimento do diagnóstico diferencial^3^. Os aspectos clínicos da lesão se manifestam de acordo com sua profundidade e localização. A coloração pode variar do vermelho ao roxo, as bordas são definidas, a consistência pode ser elástica ou fibrosa, com superfície lisa ou irregular^3^^,^^12^^,^^14^. O tamanho é variável de milímetros a centímetros^3^. Apresenta crescimento progressivo e sangramentos inesperados de difícil controle, quando ocorrem traumas no local da lesão^11^. Além disso, dor, ulceração, problemas na função mastigatória, obstrução das vias aéreas e deformação tecidual podem estar presentes^2^.

Para a escolha do tipo de tratamento dos hemangiomas, alguns atributos devem ser levados em consideração, como tamanho, localização e duração da lesão, idade do paciente, hemodinâmica, através da observação do fluxo sanguíneo, além da viabilidade da técnica^3^^,^^15^. As opções terapêuticas incluem remoção cirúrgica, administração de corticosteroides sistêmicos, crioterapia, embolização, radioterapia, laserterapia e ESCT^7^. No presente trabalho, optou-se por uma associação da laserterapia de baixa intensidade com ESCT.

A FTBM, também conhecida como terapia a *laser* de baixa intensidade, utiliza baixos níveis de luz no comprimento de onda vermelha ou infravermelha. Atua nos tecidos lesionados, promovendo a cura, remodelação e/ou redução da inflamação, o que induz a analgesia^16^. Entre as vantagens da terapia a *laser* relatadas na literatura, estão ausência de efeitos colaterais, tratamento seguro de pacientes comprometidos sistemicamente, abordagem pouco invasiva, não dolorosa e realizada em sessões curtas^8^^,^^10^^,^^17^. A ação analgésica da laserterapia é observada neste caso, em que a paciente apresentava dor aguda e, após o procedimento, relatou melhora significativa desse sintoma.

A ESCT é uma técnica efetiva para o tratamento de hemangiomas^3^^,^^15^, porém não há protocolos específicos^13^. O agente esclerosante pode ser um derivado do ácido oleico, com propriedades hemostáticas. Sua ação ocorre mediante uma resposta inflamatória, envolvendo a troca do componente vascular por um tecido fibroso^3^^,^^15^. O oleoato de menoetanolamina 5% mostrou resultados satisfatórios, com boa regressão da lesão, mínima morbidade e efeitos adversos.

As lesões vasculares em cavidade oral, como os hemangiomas, devem ter suas origens elucidadas. Os cirurgiões-dentistas devem estar aptos a reconhecer, diagnosticar, tratar e realizar os encaminhamentos adequados, quando necessários. A terapêutica com ESCT se mostra bastante efetiva, pouco invasiva, segura e com resposta rápida. A associação com a FTBM pode ser aplicada com o intuito de promover analgesia e iniciar o processo reparativo.
